# Age-related Gendered Diminishment: toward understanding and interventions for a common psychological experience in post-midlife women

**DOI:** 10.3389/fpsyg.2025.1535145

**Published:** 2025-04-01

**Authors:** Gail Saltz

**Affiliations:** ^1^NewYork-Presbyterian Hospital, New York, NY, United States; ^2^Weill Cornell Medical College, Cornell University, New York, NY, United States

**Keywords:** Age-related Gendered Diminishment, ageist sexism, mid-life, women’s mental health, generativity, invisible woman syndrome

## Abstract

Building from clinical observations with women at midlife and beyond, Age-related Gendered Diminishment (AGD) is presented as an integrated construct for a common psychological experience characterized by feelings of invisibility and inconsequentiality for women after midlife. Etiological contributors are suggested and aligned to existing literature describing frequent psychological challenges of women at this stage of life; potential evolutionary and social roots are discussed; and the question of why this complex of symptoms remains under-recognized in mental health contexts is evaluated. Finally, therapeutic interventions aimed at the construction of potential generative identities are proposed with a call for further research to better understand and address this phenomenon.

## Introduction

Everyone ages, but not everyone experiences aging in the same physical and psychological ways, and the spectrum of subjective mental and emotional experiences associated with aging can range from debilitating to empowering. Moreover, a person experiencing multiple effects of aging (e.g., changes in appearance, shift in physical fitness, deviations in cognitive functioning) may not have a homogenously positive, neutral, or negative experience with those symptoms. As a relatively benign example: A person may love their newly graying hair but detest the wrinkles around their eyes.

For all people, eventually, the experience of aging will exert adverse effects on physiological health. Recently, there has been increased attention to the myriad links between biological aging, which is the strongest risk factor for many illnesses, and physiological health, but there remain many questions as to the ways in which aging shifts psychopathology (Han et al., 2019) and how this connection might manifest. For instance, more than half of all cases of depression onset in later life (Fiske et al., 2009) and there appears to be a strong connection between late-onset depression and cerebrovascular compromise in elderly patients (Culang-Reinlieb et al., 2011). It is not yet clear, however, how putatively normal brain aging—that which is free from dementia but nonetheless associated with structural changes that impact brain function in early mid-age (Fjell and Walhovd, 2010)—impacts psychological well-being.

Whatever the causes, psychologists intuitively and experientially understand that both men and women experience changes in psychological health at and beyond post-mid-age. Differing social pressures, hardships related to gendered expectations, and biological factors may impact the ways in which people commonly experience these changes. For instance, it has been suggested that women have greater exposure to risk-factors for mental illness after mid-age, while men may be more vulnerable to these factors (Kiely et al., 2019). Thus, knowledge of the ways in which men and women differentially experience psychological hardships post mid-age may be useful for tailored treatment.

Prior research has connected feelings of inconsequentiality among post-mid-age women to a lack of representation in the media, a shift away from perceptions of sexual desirability, feelings of being ignored in social spaces, the experience of being seen only through stereotypical grandmotherly roles, and increasing encounters with patronization (Westwood, 2022). These themes of experience at the nexus of ageism and sexism strongly align to my experiences providing psychiatric and psychoanalytic care for men and women, the latter of whom appear to very commonly endure an experience that has sometimes been referred to as “invisible woman syndrome” (Dennis, 2022). However, the lay nomenclature might be problematically colloquial (Dobson, 2003) and pathologizing (Sholl, 2017). Moreover, the term suggests a solely intrinsic condition rather than a consequence of external forces, and while the holistic causes of this experience may indeed include biological predispositions (as will be discussed in this article), the majority of existing research on this phenomenon suggests social etiologies (Westwood, 2023; Lau, 2023).

Diagnostic labels can exert powerful influence on people’s willingness to seek treatment, hence many recent efforts to change the formal names of some conditions (Sartorius et al., 2014). It has further been shown that diagnostic labels may be a “mixed blessing” for people experiencing marginal levels of mental ill-health, sometimes in beneficial ways (such as promoting help-seeking, empathy, and support) but often in detrimental ways (such as undermining perceived agency and expectations that problems can be overcome) (Altmann et al., 2024). Efforts to give name to a psychological experience, particularly one that is widely and detrimentally endured, should be taken with care.

I therefore propose the term Age-related Gendered Diminishment (AGD) without pretense or expectation of immediate or even eventual widespread adoption, albeit with the hope of moving forward long overdue conversations about how to best identify and provide care for individuals who experience a suite of symptoms common to women as they transition through and beyond midlife, often encompassing feelings of invisibility, marginalization, and reduced social relevance.

## The common constellation

In 31 years as a psychiatrist and psychoanalyst, I have rarely seen two patients with the exact same arrangement of indicators, and even those with common diagnoses exhibit widespread manifestational variances. In those I have come to see as afflicted by AGD, however, there appears to be a common constellation including:

### Psychological indicators

Feelings of marginalization: A pervasive sense of being overlooked or undervalued, especially in settings where a person once felt acknowledged or validated (e.g., the workplace, social events).
Diminished self-worth: A decreased sense of confidence or self-esteem tied to societal attitudes about aging, often exacerbated by media portrayals.
Identity crisis/Identity uncertainty: Increased feelings of confusion about belonging, beliefs, and purpose often related to values, careers, relationships, or life roles.
Increased self-consciousness: Heightened awareness of physical appearance and aging signs, leading to insecurity about wrinkles, gray hair, or other age-related changes.
Morbid thoughts: An increase in contemplations related to mortality, aging, and existential concerns, often manifesting as fears about health decline, dependence, or becoming a burden. These thoughts may also involve grieving lost youth or opportunities, regrets about prior paths not taken and intensifying anxieties about the future.
Loneliness or isolation: A growing feeling of disconnection from social groups or a perception that they are less sought after for advice, company, or conversation.

### Social and parasocial indicators

Experiences of dismissal: An increased perception that opinions, expertise, or suggestions are ignored or dismissed—not only by men but by women as well—in professional, civic settings and also family and social settings, and particularly in ways that do not seem to have occurred earlier in life.
Reduced social invitations: A noticeable decline in invitations to social gatherings or events, reinforcing a sense of fading social presence, decreased importance to others, and the self-perception that one is interesting to others.
Lack of representation: A perception that few relatable role models or representations exist in media, cinema, fashion, and advertising.

### Behavioral indicators

Withdrawal: Gradual disengagement from social activities that previously brought fulfillment, often due to a perception of no longer “fitting in” and sometimes without concrete evidence that one is not, in fact, welcome in such social settings.
Increased effort in appearance: A drive to look younger or to adopt specific styles and routines to appear more relevant, valued, or “visible,” sometimes at great expense and often with a sense of playing “a losing game.”
Overcompensation in professional/family settings: An increased drive to prove value through extra effort, expertise, or support, attempting to counteract feelings of invisibility, sometimes resulting in strained relationships with colleagues and loved ones.

### Emotional indicators

Frustration or resentment: Feelings of frustration or anger, sometimes toward ageist societal attitudes but often toward oneself for “not doing enough” to stay visible and relevant.
Sadness or mourning: Grief over losing a sense of societal recognition, often accompanied by a sense that a version of oneself has died.

It is important to note that this constellation is based on my own anecdotal clinical observations of patients I see in my practice, which includes some diversity of ages, sex, gender expressions, ethnicities, and socio-economic statuses, but which in no way is a representative sample of any population at any scale. It is thus offered as a starting point for further discussion with other practitioners and researchers, with the expectation that any ultimate definition that seeks to describe this constellation as a construct would be much transformed by such communication and interrogation.

## Prevalence

Starting early into my practice, I was deeply struck by the number of women coming to me for assistance with common psychiatric diagnosis such as Major Depression, Dysthymia, Generalized Anxiety Disorder, Social Anxiety Disorder, or Adjustment Disorder, who also self-reported feelings of invisibility and inconsequentiality aligned with what I have described above as the common signs of AGD. Among those symptoms, one of the most prevalent was a loss of, or uncertainty about, identity, particularly when previous iterations of the perception of the self were connected to subjective ideas of being young, attractive, athletic, and energetic.

Indeed, the pervasiveness of this experience is reflected in an open-ended survey of more than 1,800 middle-aged women across the United States in which the experiences of irrelevance and invisibility were among the most common themes despite the fact that none of the survey’s questions were directly related to those subjects (Hofmeier et al., 2017). In a less formal but nonetheless revealing survey of more than 1,000 users of a website dedicated to issues facing mothers and grandmothers, 70 percent of respondents agreed that women “become ‘invisible’ as they get older.” The average age at which respondents estimated this experience happens was 53, although those who agreed that it had already happened to themselves estimated it began 5 years earlier than that, on average (Gransnet, 2016).

Other qualitative research efforts have affirmed that women in their post-midlife years frequently experience diminished visibility in both professional and social spheres. This includes a study including interviews with 44 women aged 50 to 70 years in which it was found that experiences with ageism and sexism intersect to influence heightened feelings of marginalization, often exacerbated by societal pressures to adhere to youthful beauty standards (Clarke and Griffin, 2008). Another study including interviews with 13 women aged 60 to 69 found that concerns about visibility were among several key driving forces for dietary decision-making (Liechty, 2012).

The seeming ubiquity of this experience is important in the context of care, as the notion that an experience must be “abnormal” to constitute a disease or disorder is powerful (Scully, 2004). Indeed, it was not surprising to learn that many of my patients previously had either implicitly concluded, or explicitly been told, that what they were going through was a normal part of aging to be endured, not a condition that, having already adversely affected their “state of complete physical, mental and social well-being,” per the preamble to the constitution of the World Health Organization (WHO, 1946), was worthy of therapeutic care.

## Evolutionary and social underpinnings

The nearly exclusive impact of AGD on women may in part be understood through an evolutionary lens. Evolutionary psychologists have suggested that women’s social value has historically been tied to their reproductive potential, which declines with age as a factor of biology, and some research has suggested that characteristics signaling reproductive capacity are valued more by males than by females across cultures (Buss, 1989). The biological reality that women are increasingly unlikely to be fertile as they experience aging, and implication that this impacts social standing, was reinforced in a study suggesting positive correlations between late estrogen, which plays a key role in female reproductive health, and subjective ratings of femininity, attractiveness, and health as estimated by both male and female evaluators (Smith et al., 2006), affecting perceived social relevance.

Social constructs around aging further amplify these likely evolutionary influences. The “Double Standard of Aging,” for instance, posits that men are often rewarded with increasing status as they age while women are devalued (Sontag, 2018). Although this double standard was described more than half a century ago, its abiding prevalence has been repeatedly reaffirmed over the years in popular media (Bazzini et al., 1997; Lauzen and Dozier, 2005); social acceptance of “appropriate” expressions of gender and sexuality (Pickard, 2022); and employment (Beery, 2021; McLaughlin and Neumark, 2024).

Societal expectations further reinforce the notion that women must maintain youthfulness to remain relevant. Todd Nelson’s seminal work on ageism, for instance, repeatedly emphasizes that women face higher societal pressure to avoid showing signs of aging, in particularly because of the conflation of beauty and worth, leading many women to feel disregarded when they no longer match youthful ideals (Nelson, 2015).

## Generativity-based therapy

Despite its significant impact on mental health and quality of life, AGD (however named) remains under-recognized within mental health frameworks. Recognizing AGD’s psychological impact could pave the way for more targeted interventions, ultimately improving the quality of life for women experiencing these symptoms. This will, of course, require far more expansive study, particularly as we seek effective treatment protocols.

In the meantime, when I encounter female patients who are at or approaching later-mid-life, and who exhibit one or more of each of the psychological, social/parasocial, behavioral, and emotional symptoms described above, one of the approaches I am taking—and which seems to have a profound effect on their psychological wellness—is a psychoanalytic approach aimed at investigating the interaction of conscious and unconscious drivers of AGD. This approach comprises a sequence of exercises informed by my observation that, in many women, identity confusion and crisis are omnipresent during this phase of life, precipitating a need for redefinition of one’s values and roles.

While there is no “correct” set of values or “right” role for all women of a certain age, generativity is often viewed as a common and critical midlife developmental stage that exerts a positive effect on psychological health (Keyes and Ryff, 1998). Generativity is typically defined as a concern for establishing and guiding the next generation, which can be expressed through parenting, teaching, mentoring, and involvement in social organizations. The concept was first introduced by psychoanalyst Erikson (1950, 1982), whose theory of psychosocial development set the groundwork for the idea of moratorium-achievement cycles in identity development, in which a person’s identity statuses shift between exploration and resolution (Stephen et al., 1992). This concept was also expanded on by McAdams and de St Aubin (1992), who developed a multidimensional model of generativity, identifying key components such as generative concern, commitment, action, and narration, to support the assessment of generativity in people’s lives. Although not explicitly intended to be a structure for mental health care, this assessment is often used as an initializing framework for intervention. The explorational process I have followed with my patients is intended to help them identify latent and potential generative actions than can be in woven into a generative identity. We then work together to assess whether that identity is authentically felt and, if so, what impacts it has on feelings of marginalization and diminishment.

While each patient’s experience and exploration is different, in my practice, their journey to understanding and addressing AGD often moves through the following sequence:

### Identifying a feeling state story

Patients are guided through the identification or construction of a personal narrative that they presently feel provides the most sensical explanation for their experience with AGD. I assure them that we are not yet trying to uncover the “real” story; whatever explanation feels most real to them is the correct story at this juncture.

### Census of joys and desires

There are many ways to be generative. In my experience, however, there is little psychological benefit (and indeed, there may be deep harm) in actions that are definitionally generative but not authentically joyful and meaningful. Thus, in this part of the sequence, I work with my patients to construct a list of their greatest joys and deepest desires—irrespective of whether anything on that list is patently generative. Importantly, this list often includes things they already do that result in pleasure, hope, and feelings of fulfillment (We can often build upon those experiences, extending them into somewhat different directions in order to create more meaningfully generative experiences).

### Aligning joys with needs to create purpose

Ultimately, I have found, a self-constructed perception that one is generative—of such a degree that this perception has a counteracting effect on AGD—is difficult if not impossible to maintain if it is not met by evidence of an actual need that is being fulfilled. Internally the motivator of having meaningful purpose is ultimately needed to sustain ongoing activity that decreases AGD symptomatology. So, in this part of the sequence, I work with my patients to identify joys and desires that align in some way with an existing need.

### Do and assess

The next part of the sequence requires action. Having aligned a joy or desire to a need, I ask patients to take steps to engage in generative activity related to that nexus. After each incremental act, I ask them to reflect on whether they feel more visible, respected, or content with life (Positive perceptions are reasons to continue onward to assess whether the cause and effect relationship is sustaining; neutral perceptions are generally an indicator that another nexus of joy and need should be sought; negative perceptions are exceedingly rare).

### Validation

Individuals who are acting in generative ways but who do not perceive impact and respect in addition to a sense of internal meaningful purpose are more likely to disengage in those actions. But a lack of perception of impact and a lack of actual affect are two very different things. One problem in this regard, however, is that women in general—and older women in particular—are often hesitant to actively seek validation. In this part of the sequence, I work with patients to overcome this disinclination, encouraging them to seek out the feedback that is so vital to the next step of this journey.

### Telling a new self-story

In this part of the sequence, patients are guided through the construction of a personal life narrative that explicitly includes their generative acts and impact on the world. If this narrative feels authentic, both immediately and over time, it can be said that they have succeeded in identifying a generative identity that can be folded into their intersectional self, and which often appears to take on a substantial role in the emerging self-identity.

## Discussion

The studies upon which this perspective article is based, and the author’s observations of differing constellations of indicators of male and female patients in post mid-life, are limited assessments of the needs of a specific, help-seeking population. While these assessments allow for the drawing of certain inferences, it cannot not be concluded that the totality of adverse psychological experiences of women are more or less profound compared to men. Men appear to face some psychological hardships that are more common to their gender, while women appear to face some psychological hardships that are more common to theirs, among which it is suggested here that the experience I have described as AGD (however ultimately termed) may be quite common, although this observation, based on clinical experience, would be better developed with comparison studies. Such studies would be beneficial in expanding upon the hypothesis that AGD is a common but largely unaddressed experience that significantly impacts the mental health of post-midlife women, and may explore the unanswered question of how AGD is experienced by trans, non-binary, intersex, and other gender expressions and identities.

While the constellation described here as AGD is common, it is by no mean the only experience that women have with aging at and beyond midlife. Indeed, it has been shown that older women often report better emotional well-being than their younger counterparts, perhaps related to increased acceptance and reduced anxiety about aging (Barrett and Toothman, 2016). Prior research has also indicated that physiological health status is not the main driver of psychological well-being in later life for women; relationships between feeling states, ego integrity, despair, concern about aging, health, and psychological well-being are parts of a comprehensive set of factors impacting how women feel about their lives as they approach 50 and beyond (Newton et al., 2022). It is thus possible that a woman may experience many, or even all the indicators described as indicative of AGD while still having a subjectively positive holistic experience with aging. Likewise, it is possible that a person may engage in the process of creating and adopting a generative identity without positive therapeutic effect.

Also worth much further discussion is the contention here that recognizing AGD as a distinct mental health issue would not only validate the experiences of millions of women but also encourage the development of supportive interventions, among which generativity-based therapies appear to hold promise as a way to help women reclaim a sense of visibility and self-worth. Future research would obviously be needed, however, to explore the efficacy of such interventions and seek to further understand the complex social, evolutionary, and psychological factors that contribute to AGD.

## Data Availability

The original contributions presented in the study are included in the article/supplementary material, further inquiries can be directed to the corresponding author.
